# 2237. Outcomes of Metronidazole Dosed Every 12 Hours Versus Every 8 Hours

**DOI:** 10.1093/ofid/ofad500.1859

**Published:** 2023-11-27

**Authors:** Maggie Box, Kelly Duong, Kyle Molina

**Affiliations:** Scripps Memorial Hospital La Jolla, La Jolla, California; Scripps Health, San Diego, California; Scripps Health, San Diego, California

## Abstract

**Background:**

Metronidazole (MTZ) is a mainstay treatment for anaerobic infections. Due to its pharmacokinetic and phamacodynamic properties, it can be dosed at 8 -, 12-, or 24-hour intervals. MTZ’s adverse effects depend on the cumulative dose, permitting less frequent administration. Less frequent dosing intervals may save nursing and pharmacy resources and improve patient compliance. Further, drug shortages of IV MTZ have prompted efforts to reduce utilization to preserve the availability of therapy. The objective of this study was to compare clinical outcomes of patients who received MTZ every 8 hours to every 12 hours.

**Methods:**

This multicenter, retrospective study was conducted at Scripps Health from 1/1/2022 - 12/31/2022. Hospitalized adult patients who received MTZ, dosed at 500 mg every 8 hours or every 12 hours, for > 5 days for a pulmonary, intra-abdominal, or skin source were included. Patients were excluded if they received anaerobic antibiotics for > 72 hours prior to MTZ or concurrent to MTZ, or were pregnant, transitioned to hospice, or received MTZ for < 75% of the total therapy duration. The primary outcome was clinical cure and all-cause mortality within 30 days. Secondary outcomes were escalation of therapy, 30-day readmission, and duration of therapy.

**Results:**

A total of 107 patients were included with similar baseline characteristics per group. The predominant infection source was intra-abdominal (55.1%), followed by skin (29.1%) and pneumonia (12.1%). An anaerobic organism was isolated in 16.8%, and source control was performed in 47.7%. All patients received concurrent antibiotics. Comparing every 8-hour (n = 52) versus every 12-hour (n = 55) MTZ, there was no difference in clinical cure (94.2% vs. 100.0%, p = 0.071) or all-cause mortality (1.9% vs. 0.0%, p = 0.301), respectively. For secondary outcomes, there was no difference in escalation of therapy (3.8% vs. 1.8%, p = 0.525) or 30-day readmission (28.8% vs. 25.5%, p = 0.693) between every 8 hours and every 12 hours. Total duration of antibiotics was similar between groups (14.2 vs 13.0 days, p = 0.541).

**Conclusion:**

There was no difference in clinical cure, 30-day all-cause mortality, 30-day readmission, or escalation of therapy among patients receiving every 8 versus every 12-hour MTZ therapy.

**Disclosures:**

**All Authors**: No reported disclosures

